# Peri-procedure efficacy and safety of one-stop hybrid surgery for the treatment of brain arteriovenous malformations: A single-center preliminary experience

**DOI:** 10.3389/fneur.2022.1052882

**Published:** 2022-11-04

**Authors:** Wei Fang, Zijian Yang, Yufeng Liu, Jia Yu, Peng Sun, Zhenwei Zhao, Yue He, Tao Zhang, Jianping Deng

**Affiliations:** ^1^Department of Neurosurgery, Tangdu Hospital, Air Force Medical University, Xi'an, China; ^2^Tongji Medical College, Tongji Hospital, Huazhong University of Science and Technology, Wuhan, China

**Keywords:** cerebral arteriovenous malformation (AVM), endovascular treatment, hybrid surgery, intraoperative angiography, microsurgical treatment

## Abstract

**Background:**

Some deficiencies and shortcomings in treatment strategies of brain arteriovenous malformation (bAVM) remain. It is worth exploring whether the one-stop hybrid surgical platform can play a positive role in the treatment of bAVM.

**Objective:**

This study investigated short clinical and angiographic results of one-stop hybrid surgery for the treatment of bAVM.

**Methods:**

All patients with bAVM treated with one-stop hybrid surgery were reviewed from February 2017 to December 2021. Data including demographic information, clinical conditions, characteristics of AVM, procedure details, and clinical and angiographic results were collected.

**Result:**

In total, 150 cerebral bAVM patients received one-stop hybrid surgery; among them, 122 received surgical resection assisted by intraoperative DSA, and 28 were treated with combination surgical resection and endovascular embolization. Complete angiographic obliteration of the AVM was achieved in 136 patients (90.7%), and procedure-related death and neurological deficit rates were 7.3%. Of all relevant variables, logistic regression analysis showed that the Spetzler & Martin (S&M) score was the only factor related to the cure rate (*P* < 0.001) and endpoint complication rate (*P* = 0.007).

**Conclusions:**

In our preliminary experience, one-stop hybrid surgery for the treatment of brain AVMs achieves a high angiographic total occlusion rate, with acceptable peri-procedure morbidity and mortality. For S&M 4 and 5 lesions, more cases and further study are needed to investigate the effects and safety of hybrid surgery.

## Introduction

bAVMs are a main cause of cerebral hemorrhage in young patients (1–4). The annual risk of intracranial hemorrhage is reportedly between 2 and 4%, with an overall mortality after intracerebral hemorrhage of 18% (5–7). Incomplete treatment of AVM is ineffective and might increase the risk of hemorrhage (7–9). Therefore, complete elimination of the lesion is considered to be the most efficient method to reduce hemorrhage risk (10–12). In general, curative treatment for AVM should be considered the ultimate goal in most cases.

The traditional modality of treatment for bAVMs includes radiosurgery, microsurgical resection and embolization. Stereotactic radiosurgery has poor efficacy for large AVMs (13); patient bleeding may still occur or even increase during the interim (14). Microsurgical resection is recommended for low Spetzler & Martin (S&M) grade lesions due to its high cure rate and low surgical morbidity and mortality (15–18). However, microsurgery is not recommended for high-grade AVMs, especially for Grades IV and V, as its complication rate is as high as 30% (19). Although endovascular embolization has always been included as part of the multimodal treatment strategy, it is not considered a promising method. Preoperative embolization seems to facilitate surgical removal (20, 21). Only in recent years has curative embolization gradually become the goal of clinical pursuits, with strictly selected cases (22). Nonetheless, the complication rate is not as low as expected (23).

As each modality has its own advantages and shortcomings, it is recommended that AVMs be treated by multidisciplinary groups, including neurosurgeons and neuroradiologists. One-stop hybrid surgery is a relatively new modality for the treatment of bAVM that combines endovascular techniques with microsurgical techniques, enabling them to complement each other to achieve the maximum clinical benefit (12, 24–27). However, there are few reports with limited cases to examine their value and benefits. In this study, we summarize consecutive AVM patients treated with hybrid surgery in a high-volume tertiary medical center to investigate the short-term effect and safety of hybrid surgery for the treatment of brain AVM.

## Materials and methods

The prospective database of cerebral vascular diseases at the Neurosurgery Department, Tangdu Hospital was retrospectively retrieved. From February 2017 to December 2021, all patients with cerebral AVM treated with one-step hybrid surgery were selected. All data including demographic information (sex and age), clinical conditions (presentations, preoperative and postoperative mRS scores), characteristics of AVM (including S&M grade, AVM location, pattern of nidus [diffuse or compact], venous drainage [deep draining or not], related aneurysms), procedure details, and clinical and angiographic results were collected. The primary endpoints were the complete AVM angiographic obliteration rate immediately after surgery and procedure-related morbidity and mortality at 30 days postoperatively. Procedure-related morbidity and mortality was defined as neurologic dysfunction and death resulting from surgery. The secondary endpoint was the rate of good condition referring to mRS < 2 at 30 days postoperatively, any nonprocedure-related complications and procedure-related complications excluding neurodeficit or death.

This study was approved by the local ethics committee.

### Hybrid surgery procedure

The equipment used for the hybrid operation is mainly composed of a C-arm angiography system (Siemens Artis Zee Biplane) and a surgical microscope (Zeiss). Intraoperative radiolucent equipment included a carbon head holder and head pins (Doro Germany) to optimize intraoperative computed tomography CT and DSA imaging quality.

All procedures were carried out under general anesthesia with intubation. Two kinds of surgical protocols were implemented: surgical resection assisted by intraoperative digital subtraction angiogram (iDSA) and surgical resection combined with pre- or intrasurgical embolization. All procedures started with the introduction of a 6-French femoral sheath. For presurgical embolization, heparinization with 3,000 U heparin and reversed with protamine immediately after embolization and before surgical resection was performed. According to the AVM location, different operating positions were used for patients treated with presurgical embolization or surgical resection directly. For the supine position, only a single femoral approach was required because we could easily adjust the position of the angiographic catheter during surgery. For the prone position, both femoral approaches were sometimes necessary for lesions with multiple arterial feeders belonging to different circulations. A guiding catheter or angiographic catheter was placed in the target artery in advance. For intraoperative DSA and intrasurgical embolization, the catheter was continuously flushed with pressured saline. All imaging, endovascular manipulations and surgeries can be performed sequentially or in parallel, without the necessity of moving the patient (24). For iDSA, two or more clips were typically used as markers to accurately locate the residual AVM lesion. For all cases, surgical outcomes were ascertained by 2D or 3D iDSA before craniotomy closure. After closure, 2D and 3D DSA and Dyna CT were performed routinely to confirm total obliteration of the AVM and to exclude unexpected hemorrhage. Intra- and postoperative DSA images were assessed by two neurosurgeons and neuroradiologists.

**Figure 1:**
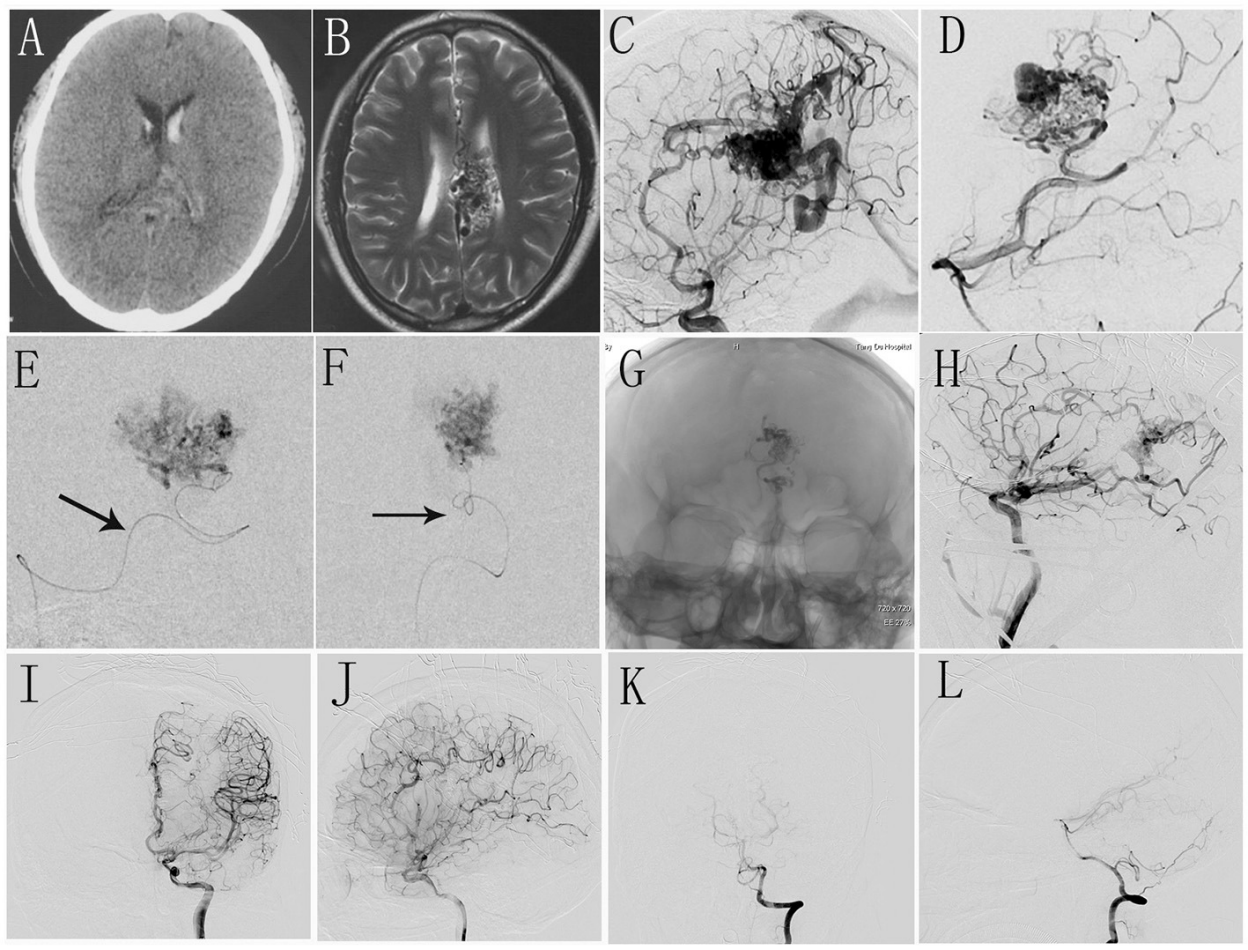
**(A,B)** Preoperative CT and MR scan shows intraventricular hemorrhage and malformed vascular mass in corpus callosum. **(C,D)** Intraoperative DSA shows AVM located in corpus callosum with dual blood supply from anterior cerebral artery and posterior cerebral artery. **(E,F)** Selective angiography shows the deep feeder artery and embolization was performed. **(G)** The morphology of Onyx glue under X-ray view. **(H)** Intraoperative DSA shows the residual AVM nidus. **(I–L)** The immediate postoperative DSA shows complete occlusion of AVM.

The decision of whether to embolize the AVM before surgery was discussed by the neurosurgeons and neuroradiologist together. The principle of embolization is to reduce surgical risk. The targets of embolization were as follows: 1. Deep feeder arteries are difficult to reach and control during surgical procedures; 2. hemodynamics-related aneurysms 3. AVM compartment adjacent to eloquent areas. Intra- and presurgical embolization was performed when it was considered that the lesion could be easily occluded, with little risk. All embolizations were performed using low-density Onyx (Onyx 18, Micro Therapeutics Inc., Irvine, California, USA) (Figure 1).

### Postoperative care and follow-up

All patients were monitored in the neurosurgical intensive care unit for at least 24 h after the operation. For larger lesions (diameter more than 3 cm), blood pressure was kept at 30 mmHg lower than baseline. A CT scan was routinely performed within 24 h postoperatively. Clinical follow-up was administered at 30 days after surgery.

### Statistical analysis

Analyses were performed with SPSS 26 for relative factors with cure rate and procedure-related morbidity and mortality. Possible factors included sex (male and female), age, preprocedure mRS score, characteristics of AVM and presentation. The chi square test was used for univariate analysis, and the positive factor was analyzed with logistic regression multivariate analysis. The limit of significance was set at 0.05.

## Results

### Baseline characteristics

From February 2017 to December 2021, 150 patients with brain AVM were treated by hybrid surgery. As detailed in Table 1, there were 100 men and 50 women, with a mean age of 31.3 ± 15.6 years, ranging from 6 to 69 years. Cerebral hemorrhage was the main presentation in 115 cases (76.7%); other manifestations, such as epilepsy in 13 cases (8.7%), headache in 13 (8.7%), neurological dysfunction in 5 (3.3%) and no symptoms in 4 (2.7%), also occurred. The mRS distribution was as follows: 33 cases of 0 (22.0%), 80 cases of 1 (53.3%), five cases of 2 (3.3%), 11 cases of 3 (7.3%), 14 cases of 4 (9.3%), and seven cases of 5 (4.7%). A good clinical status (mRS = 0 or 1) was observed in 113 patients (75.3%).

**Table 1:** Patient's characteristics.

**Baseline characteristics**	***n* = 150 (100%)**
Age (years) (mean ± SD)	31.3 ± 15.6
Gender	
Male	100 (66.7)
Female	50 (33.3)
Sex ratio	21
Clinical manifestation	
Hemorrhage	115 (76.7)
Epilepsy	13 (8.7)
Headache	13 (8.7)
Neurological dysfunction	5 (3.3)
Atypical manifestation	4 (2.7)
Preoperative mRS	
0	33 (22.0)
1	80 (53.3)
2	5 (3.3)
3	11 (7.3)
4	14 (9.3)
5	7 (4.7)
AVM location	
Supratentorial	139 (92.7)
Frontal	32 (21.3)
Temporal	42 (28.0)
Parieto-occipital	29 (19.3)
Ventricular and periventricular	18 (12.0)
Deep (sylvian insular, basal ganglial and thalamic)	18 (12.0)
Subtentorial	11 (7.3)
Cerebellar	9(6.0)
Foramen magnum	2 (1.3)
Venous drainage	
Deep	62 (41.3)
Superficial	88(58.7)
Hemodynamics related aneurysms	
Yes	10 (6.7)
No	140 (93.3)
S&M grade	
1	34 (22.7)
2	60 (40.0)
3	34 (22.7)
4	21 (14.0)
5	1 (0.6)
Other features	
Compact	113 (75.3)
Diffuse	37 (24.7)

According to S&M classification, there were 34 patients with Grade I (22.7%), 60 with Grade II (40.0%), 34 with Grade III (22.7%), 21 with Grade IV (14.0%), and 1 with SM Grade V (0.6%). Sixty-two cases (41.3%) involved deep venous drainage and 88 (58.7%) only superficial venous drainage. A total of 139 AVMs were supratentorial (92.7%): frontal (32 cases), temporal (42 cases), parieto-occipital (29 cases), ventricular and periventricular (18 cases), and deep (18 cases). Eleven subtentorial lesions included nine cases in the cerebellum and two cases in the foramen magnum. Among other features, 113 patients (75.3%) had compact lesions, and 37 patients had diffuse lesions (24.7%). Hemodynamically associated aneurysms were found in 10 cases (6.7%).

### Procedure characteristics

Of the 150 patients, 122 received surgical resection assisted by intraoperative DSA, whereas 28 were treated with a combination of surgical resection and endovascular embolization. Preoperative angiography was performed in 67 patients before craniotomy to help design the bone flap or before microdissection to accurately locate the nidus.

Among those treated with surgical resection under supervision of intraoperative DSA, DSA was carried out only once in 63, with the results of total AVM resection. The remaining 59 patients received at least two iDSA procedures, and most received up to 15 iDSA procedures to ascertain the surgical degree and to navigate the microdissection.

Among the patients treated both with resection and embolization, 25 received presurgical embolization and three intrasurgical embolization. For presurgical embolization, a total of 41 sessions were performed: single embolization for 16 patients, two sessions for four patients, three sessions for three patients and four sessions for two patients. For intrasurgical embolization, only one session was conducted in all three patients.

### Complete occlusion

Of the 150 patients, complete obliteration of AVM, as ascertained by 2D and 3D DSA after closure, was achieved in 136 (90.7%). Incomplete resection occurred in the other 14 patients due to different causes. In one patient, there was no opportunity to undergo the last DSA because of the anesthesia complication of malignant hyperthermia. Two patients had unexpected residuals because these two AVMs had different circulation arterial feeders, and iDSA missed the small residual. The unfeasibility of complete resection for the other 11 cases had been considered in the preoperative plan owing the deep and eloquent location; in such a situation, complete resection would bring more destruction to brain tissue, which may lead to neurological deficits. For 133 patients, complete obliteration of lesions was obtained by surgical resection, and 3 patients reached this result with intrasurgical embolization.

The chi square test showed that only the pattern of nidus (diffuse or compact) (*P* = 0.028) and S&M grade (*P* < 0.001) were significantly related to the cure rate. No relationship was found between cure rate and sex (*P* = 0.68), age (*P* = 0.42), symptoms (*P* = 0.51) or preoperative mRS score (*P* = 0.93). Further multivariate analysis with logistic regression was performed, and only S&M grade remained a significant factor (*P* < 0.001). The complete obliteration rate according to S&M grade was as follows: 100% for Grade I, 96.7% for Grade II, 91.2% for Grade III, 61.9% for Grade IV and partial removal for the only Grade V case (Table 2).

**Table 2:** Occulusion rate and procedure-related morbidity and mortality in different S&M grade.

**S&M grade**	**Complete occlusion rate (%)**	**Procedure-related morbidity and mortality (%)**
I	100	2.9
II	96.7	1.7
III	91.2	14.9
IV	61.9	19.0
V	0	0

### Peri-related morbidity and mortality

The primary endpoint occurred in 11 patients (7.3%), including three deaths and eight neurologic deficits. Among the three deaths, severe cerebral edema postsurgery was the main reason for two: in one, edema did not resolve even with decompressive craniectomy; the other refused further treatment because of a poor condition presurgery. The third patient had a good clinical status with only slight limb weakness postoperatively. However, she experienced a sudden coma 24 h later, though an emergency CT scan showed unremarkable changes. Of the eight patients with neurological deficits, six had hemiplegia, with five directly resulting from the surgical resection procedure and the other from hemorrhage during presurgical embolization. Two patients experienced aphasia and limb paresis.

Both the chi-square test and logistic regression showed S&M score (*P* = 0.007) to be a significant factor related to procedure-related neurodegeneration and death. Other factors, including sex (*P* = 0.42), age (*P* = 0.94), symptoms (*P* = 0.97), pattern of nidus (*P* = 0.20) and mRS score (*P* = 0.23), had no relationship with procedure-related neurodysfunction or death. According to the degree of SM, the primary endpoint of the surgery-related complication rate was 2.9% for Grade I, 1.7% for Grade II, 14.9% for Grade III, and 19% for Grade IV (Table 2).

There were 13 secondary endpoint complications (8.6%). One death occurred because of the anesthesia complication of malignant hyperthermia. A 10-year-old girl had severe cerebral vasospasm several days after surgery, with hemiparesis lasting more than 1 month. Two patients experienced transient symptoms of venous thrombosis and completely recovered after anticoagulant therapy. Acute epidural hematoma occurred in one patient during surgery, but there were no new clinical manifestations after evacuation. Eight cases of intracranial infection were cured with medical therapy.

At 30 days after the operation, good clinical outcomes (mRS ≤ 1) were achieved in 109 patients (72.7%). Among patients with mRS 3~5, the value returned to 0~1 in none. Only some patients with mRS 2 recovered to a better condition. Ten patients with mRS 0 or 1 deteriorated to more than mRS 1.

## Discussion

Currently, AVM is the most intractable cerebral vascular disease because of its low cure rate and high complication rate (28–30). AVM always creates a dilemma with regard to treating or not treating it, especially for unruptured lesions. The results of ARUBA added more controversy to whether treatment should be aggressive (29). Despite several modalities, including surgical resection and endovascular and stereotactic radiosurgery, for the treatment of brain AVMs, each has its own limitations. Therefore, treatment strategies are prepared by multidisciplinary teams to overcome each other's shortcomings. Theoretically, one-stop hybrid surgery has an advantage because it spatially and temporally combines two kinds of techniques to create a synergistic effect. However, there are few reported cases (12, 24–27). To our knowledge, this report involves the largest case series on hybrid surgery treatment of bAVM. We report the peri-procedural clinical and angiographic results of 150 consecutive cases managed by hybrid surgery from the first case in February 2017 to a case in December 2021. In our cohort, 11 adverse endpoint events occurred, with a rate of 7.3%, including three deaths and cases of eight neurologic deficits. At the same time, the immediate cure rate reached 90.7%. Statistical analysis showed S&M grade to be the only factor related to both cure rate (*P* < 0.001) and procedure-related neuro-dysfunction (*P* = 0.007), with significant differences between the low-scale group (S&M 1 and 2) and the high-scale group (S&M 3, 4 and 5). According to the SM degree, the primary endpoint of the surgery-related complication rate was 2.9% for Grade I, 1.7% for Grade II, 14.9% for Grade III, and 19% for Grade IV. The complete occlusion rate was as follows: 100% for Grade I, 96.7% for Grade II, 91.2% for Grade III, 61.9% for Grade IV and partial removal for the only Grade V case.

How can a modality be evaluated for the treatment of cerebral AVM?

Safety is most important. Although some transient complications may occur that would result in long hospital stays or more medication and costs, more attention should be given to permanent neurologic deficits and death, which are irreversible injuries. The endpoint complication rate of our series was basically consistent with the range reported in the literature: some systematic reviews and meta-analyses have reported surgical complication rates of ~7% (15, 31, 32). It should be noted that the adverse endpoint rate reported in the literature is largely related to patients with S&M grade I-II, whereas patients with higher grades comprised a significant proportion in our cohort. A good clinical outcome (mRS ≤ 1) was achieved in 109 patients (72.7%) at 30 days compared with 111 patients (74.1%) preoperatively. Given that most of this series presented hemorrhage and needed time to recover, this result was acceptable. Based on the above data, the neurological deterioration caused by hybrid surgery is seemingly tolerable, as unruptured AVMs have an annual hemorrhage rate of 2.2% and ruptured lesions an annual hemorrhage rate of 4.5% (33).

The second is the rate of complete occlusion. There is no evidence that partial embolization or resection can reduce the risk of hemorrhage (7). Many studies have shown that partial elimination of AVMs increases the risk of hemorrhage (7–9). On the other hand, most patients with AVM are young, with a mean age of approximately 30 years and a long life expectancy. Hence, the accumulation rate of hemorrhage and related morbidity or mortality is high enough to support intervention to treat the lesion. Therefore, for hemorrhagic or symptomatic AVMs, the best treatment strategy should be complete occlusion of the AVM. The total occlusion rate of our 150 patients was 90.7%, and for cases with S&M grades 1~3, the rate was 96.1%. Although there is a chance of recurrence during a long follow-up time, as reported by Song et al., the probability is low (27). Overall, immediate complete occlusion leads to a greater chance of cure.

Recent studies have shown that hybrid surgery is mainly applied for low S&M degree AVMs (I-II), with a low complication rate and high cure rate, the results of which are similar to those of our low S&M cases (7, 20, 26, 34). Only a small case series of Wen et al., nearly half of which were S&M 3~5, had a mortality rate of 4.5% (26). Thus, the feasibility of hybrid surgery for the treatment of high S&M grade AVM remains unknown. In our cohort, there were 34 patients with S&M III, 21 patients with S&M IV and one patient with S&M V; the total occlusion rates were 91.2, 61.9, and 0%, respectively, and the endpoint complications were 14, 19, and 0%, respectively. Compared with surgical cases, it was acceptable. Catapano et al. reported that the rate of procedure-related worsened neurological conditions was high (33.3%) for 102 S&M III AVM patients (35). Jeon's case series with 55 S&M 3 AVM patients reported 12.7% postoperative deficits, with 89.1% complete resection (36). Bervini et al. found a risk of permanent neurological deficit for unruptured brain AVM, with a rate of 14% for S&M III patients and 38.6% for S&M IV and V patients, but no cure rate was reported (16). It should be noted that most of our cases involved hemorrhage with certain neurological dysfunction, which motivated us to adopt a relatively aggressive treatment and might also increase the difficulty of surgery and increase the complication rate.

**Figure 2:**
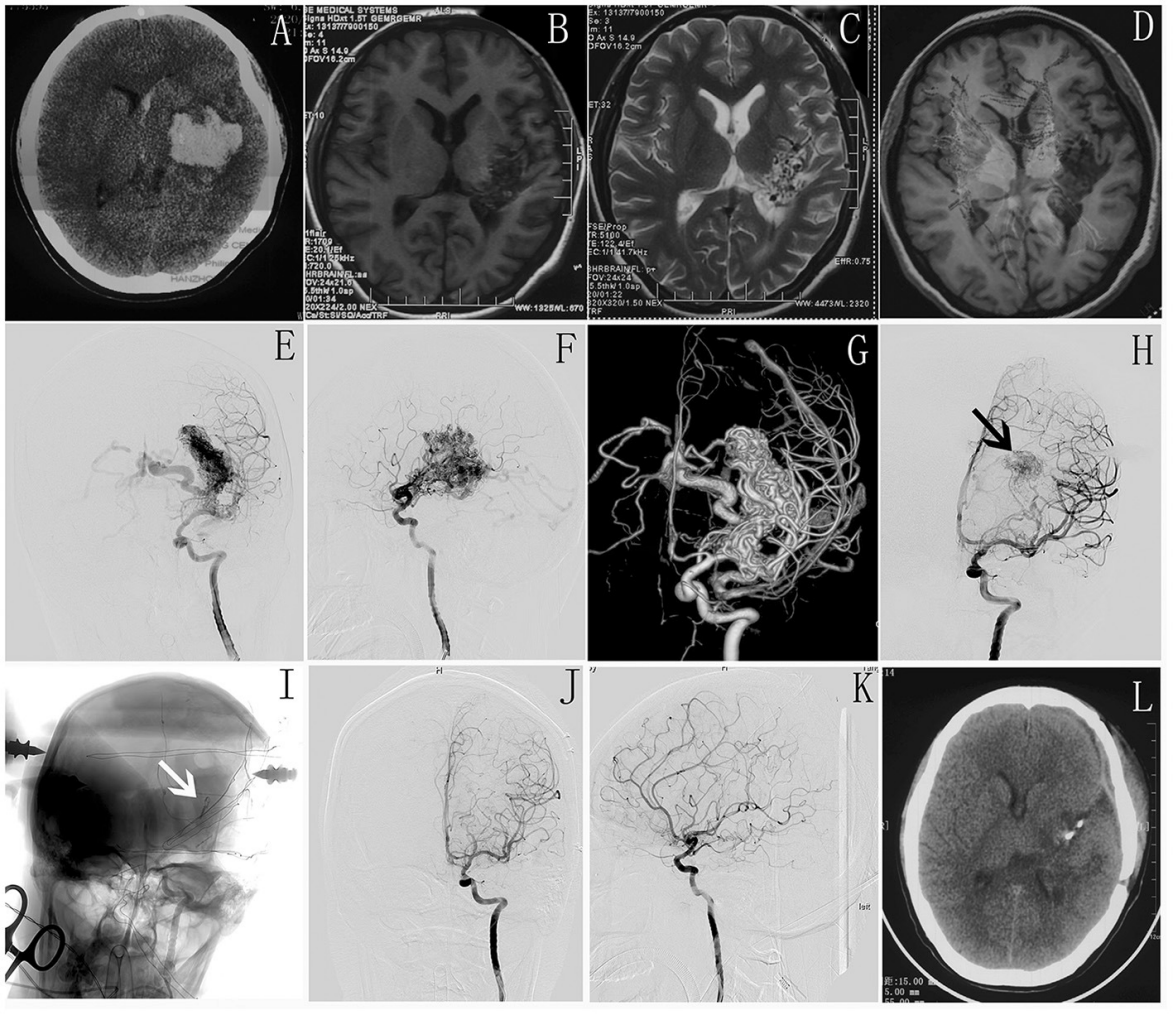
**(A)** Preoperative CT shows intracranial hematoma in left basal ganglia. **(B–D)** MR scan shows malformed vascular mass in left basal ganglia and some damage in corticospinal tract. **(E–G)** Intraoperative DSA shows AVM located in basal ganglia. **(H,I)** Intraoperative DSA shows the residual AVM nidus (white arrow) and a clipper used as markers to accurately locate the residual AVM lesion(black arrow). **(J,K)** The immediate postoperative DSA shows complete occlusion of AVM. **(L)** postoperative CT scan shows no more damage in the normal brain tissue.

The hybrid surgery suite was introduced into our center at the beginning of 2017. For AVM resection with intraoperative angiography, our preliminary experience showed that hybrid surgery has the following benefits. The immediate intraoperative angiography can improve the precision of the surgery by accurately locating and microdissecting the AVM, which allows the operator to work in close contact with the nidus, sparing brain tissue without fear of damage to normal brain tissue, especially in eloquent areas. Intraoperative angiography can also provide 2D or 3D images that show the AVM lesion remnants and navigate the surgical procedure, thereby avoiding incomplete resection (26, 37). Many of our patients underwent more than two iDSA procedures to locate the nidus remnant, avoiding excessive exploration (Figure 2). During 122 patients received surgical resection assisted by intraoperative DSA, 45 patients were with high S&M grades (3, 4 and 5) and the cure rate for this cohort was 36/45 (80%). This is a relatively satisfactory result for high-grade lesions and our further study would be to compare the cure rates between hybrid and conventional surgery.

From our experience, presurgical endovascular intervention can reduce the risk of intraoperative rupture by embolization of feeder arteries, especially deep feeder arteries that are difficult to control during the resection procedure. The embolization procedure is more important for high-grade and high-flow AVMs, as it can avoid the dilemma faced by traditional surgery. Intrasurgical embolization is a unique advantage of hybrid surgery, which can facilitate the operation and avoid damage to more brain tissue. This method was adopted in three of our cases, with the result of total occlusion. On the other hand, combining surgical resection into a single-stage procedure avoids adverse events of embolization, such as postembolization bleeding, and manages the existing complication of embolization immediately for earlier timing (34). In our department, treatment strategies are formulated according to the pathological characteristics and radiologic characteristics of cases. Preoperative embolization is routinely performed for patients with high S&M grades and suitable embolization pathways. Of the 28 patients who underwent embolization and resection, 14 had complex lesions with S&M grade III–IV; other patients with S&M grade II or I were also included after a failed attempt at curative embolization.

There are several limitations in this study. This was a retrospective study, and there was no control group, which might lead to selection bias. Because this case series study was based on our preliminary experience of the application of a hybrid operating suite, the learning curve would inevitably affect the result. An unexpected residual lesion occurred in one patient due to less experience during the initial period. Some patients had serious neurologic deficits before treatment, which may conceal damage from surgery, and the risk of hybrid surgery may be underestimated. Only a small portion of cases received pre- or intrasurgical embolization for various reasons. When reviewing the cases, we found greater opportunity for the application of embolization, which would facilitate treatment and increase the total occlusion rate, especially for high S&M cases.

## Conclusions

Our preliminary case series shows that one-stop hybrid surgery for the treatment of brain AVM can achieve a high angiographic total occlusion rate, with acceptable peri-procedure morbidity and mortality, especially for lesions with S&M grades 1~3. For S&M 4 and 5 lesions, more cases and further study are needed to investigate the effects and safety of hybrid surgery. Pre- and intrasurgical embolization is helpful to facilitate treatment and increase the complete occlusion rate. However, the best combination of surgical and endovascular techniques remains unknown. Intrasurgical angiography in the hybrid operating room is vital to improving the complete resection rate and surgical safety.

## Data availability statement

The original contributions presented in the study are included in the article/supplementary material, further inquiries can be directed to the corresponding authors.

## Ethics statement

The studies involving human participants were reviewed and approved by Ethics Committee of Tangdu Hospital. Written informed consent to participate in this study was provided by the participants' legal guardian/next of kin.

## Author contributions

WF acquired and analyzed data and wrote the first draft of the manuscript. ZY, JY, and YL performed imaging analyses and acquired related data. PS acquired clinical data. ZZ, YH, and WF contributed to the database. TZ and JD contributed to the conception and design of the study and reviewed the manuscript. All authors contributed to manuscript revision, read, and approved the submitted version.

## Conflict of interest

The authors declare that the research was conducted in the absence of any commercial or financial relationships that could be construed as a potential conflict of interest.

## Publisher's note

All claims expressed in this article are solely those of the authors and do not necessarily represent those of their affiliated organizations, or those of the publisher, the editors and the reviewers. Any product that may be evaluated in this article, or claim that may be made by its manufacturer, is not guaranteed or endorsed by the publisher.
